# Integration of Bioengineered Tools in Assisted Reproductive Technologies

**DOI:** 10.1002/adhm.202500918

**Published:** 2025-06-16

**Authors:** Aslı Ak, Edith Coonen, Ron van Golde, Erik Vrij

**Affiliations:** ^1^ Department of Cell Biology‐Inspired Tissue Engineering (cBITE) MERLN Institue for Technology‐Inspired Regenerative Medicine Maastricht University Maastricht 6229 ET The Netherlands; ^2^ Department of Obstetrics and Gynaecology GROW Research Institute for Oncology and Reproduction Maastricht University Maastricht 6229 ET The Netherlands; ^3^ Department of Clinical Genetics Maastricht University Medical Centre (MUMC+) Maastricht 6229 HX The Netherlands

**Keywords:** assisted reproductive technologies, bioengineered platforms, fertilization, implantation, in vitro models, stem cell‐based embryo models

## Abstract

Infertility affects ≈17.5% of the adult population worldwide, posing significant clinical, emotional, and socioeconomic challenges. Recent advances at the intersection of reproductive medicine and bioengineering offer promising avenues to enhance assisted reproductive technologies (ART). This review synthesizes emerging microengineered and stem cell‐based platforms designed to improve key ART stages from gamete handling and fertilization to embryo culture and implantation. State‐of‐the‐art microfluidic systems that refine sperm selection by leveraging directional behavioral responses, enhancing motility, and preserving DNA integrity is discussed. Moreover, devices for oocyte denudation and cryopreservation have been developed to mitigate cellular stress associated with conventional processing techniques. While microengineered platforms demonstrate promise in sperm sorting and reducing stress on gametes, their broader application in ART, particularly for oocyte handling and embryo culture, requires further development. The review further addresses stem cell‐based embryo models and bioengineered endometrial platforms, which aim to recapitulate the dynamic in vivo microenvironments needed for successful fertilization, embryo development, and implantation. Despite encouraging preliminary results, challenges such as scalability, reproducibility, clinical validation, and ethical considerations remain. By identifying these gaps and proposing future directions, this review considers integrating microengineering and bioengineering approaches to streamline ART procedures, ultimately enabling more personalized and effective reproductive therapies.

## Graphical abstract

A conceptual illustration depicting the collaboration between a medical professional (right) and a scientist (left). Their connection highlights the integration of scientific research and clinical practice. This representation underscores the role of emerging technologies in bridging fundamental research with applied reproductive healthcare.

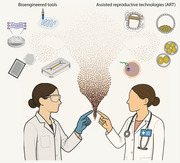

## 1 Introduction

Infertility, previously estimated to affect 186 million people globally,^[^ ^1^ ^]^ is now understood to impact ≈17.5% of the adult population according to the World Health Organization (WHO)’s 2023 report.^[^ ^2^ ^]^ These numbers underscore the scale of infertility as a global health challenge and affecting hundreds of millions across all income levels. ≈85% of infertility cases are attributed to an identifiable cause,^[^ ^3^ ^]^ yet there is no consensus on a specific rate. Male and female factors seem equally responsible for varying underlying conditions amongst which low sperm quality, blocked fallopian tubes, endometriosis, and ovulatory problems caused by polycystic ovarian syndrome, or hypothalamic dysfunction.^[^ ^4^, ^5^, ^6^ ^]^ Some studies estimate that unexplained infertility accounts for 30–38% of cases.^[^ ^7^, ^8^ ^]^ This variability highlights the complexity of diagnosing infertility and underscores the need for deeper investigation into its causes to improve current treatment options. Patients may face psychological and emotional consequences, as they experience lack of hope for future treatments, frustrations towards treatment, and depression due to previous negative outcomes.^[^ ^9^, ^10^ ^]^

The clinical focus of reproductive medicine, centered on addressing infertility and developing advanced treatments, naturally intersects with reproductive biology. Our understanding of underlying molecular and cellular processes can drive innovations that enhance assisted reproductive technologies (ART) and improve patient outcomes, as well as our ability to create more sophisticated and effective ART methods. This progress has been significantly bolstered by advancements in bioengineering and cell biology, leading to the development of innovative tools and techniques that more closely mimic natural reproductive processes. As such, several microengineered platforms have been developed over the years, including ones based on microfluidics, synthetic matrices, and surface topography.^[^ ^11^ ^]^ The overarching goal of these tools is to create a microenvironment that allows directing the number, position, behavior, and fate of the cells. Additionally, we can control the volume and content within the microfluidic systems, physical properties of the extracellular matrix (ECM), and mimic the biochemical and functional aspects of the original tissue. Recent advancements in stem cell‐based modelling, including organoids, have the potential to be of added value for obtaining a more biological complexity resembling in vivo.^[^ ^12^, ^13^ ^]^

In this review, albeit not exhaustive, we explore how microengineered platforms and advanced stem cell‐based reproductive technologies could enhance two key elements of ART procedures: fertilization and implantation. Additionally, we aimed to address a few important questions. First, what are the key intersections between two distinct fields, reproductive medicine and bioengineering, that have led to advancements, such as improved sperm selection? Second, where do we currently stand in this interdisciplinary collaboration? Are the results thus far successful, and what aspects need improvement? How can we enhance communication and knowledge exchange between these two fields to maximize progress? Finally, where is this intersection headed? Will there be a time when clinicians can routinely use bioengineered models and microengineered platforms for patient diagnosis and treatment? While exploring these questions, we also aim to identify areas where answers are still evolving.

## 2 Microengineered and Stem Cell‐Based Reproductive Tools for Fertilization

Successful fertilization in vivo is a rather complex process that relies on multiple interconnected components. Sperm motility is crucial as it enables sperm to navigate through the fallopian tube and reach the oocyte. The quality of the oocyte, encompassing factors such as chromosomal constitution, cytoplasmic maturity, mitochondrial function, and DNA integrity, is equally important. Once fertilization has occurred, embryo quality becomes paramount, influenced by the rate and symmetry of cell division, proper compaction, and blastocyst formation. Understanding and optimizing these biological components in the laboratory is essential, alongside standardizing the clinical workflow. In ART, the two most widely used techniques are: in vitro fertilization (IVF) and intracytoplasmic sperm injection (ICSI). Both involve a series of critical steps, including ovarian stimulation, oocyte retrieval, sperm selection, embryo culture and assessment, embryo cryopreservation, preimplantation genetic testing (PGT), and embryo transfer. Despite decades of advancements, these methods still rely on manual, operator‐dependent procedures, which can introduce variability and limit predictive accuracy. (**Table** **1**) To overcome these challenges, researchers are exploring automated and precision‐focused solutions.

**Table 1:** Current standard practices and limitations of clinical steps in ART (IVF and ICSI).

Clinical Step	Current Standard Practice	Limitations / Challenges
Sperm selection	Manual selection (motility and density‐based) and visualization	Open to subjectivity, excluding epigenomics and DNA integration of sperm^[^ ^14^ ^]^
Oocyte denudation	Hyaluronidase to dissolve the extracellular matrix of cumulus cells	Risk of mechanical stress, (prolonged) use of hyaluronidase leads to decreased fertilization rate^[^ ^15^ ^]^
Fertilization	IVF or ICSI	Inconsistent outcomes,^[^ ^16^ ^]^ risk of mechanical stress to gametes (especially during ICSI), risk of polyspermy^[^ ^17^ ^]^
Embryo culture	Static in vitro culture	Suboptimal microenvironment (lack of dynamic culture),^[^ ^18^ ^]^ risk of mechanical stress,^[^ ^19^, ^20^ ^]^ and use of mineral oil negatively impacts blastocyst development^[^ ^21^ ^]^
Embryo assessment	Morphological grading and time‐lapse imaging	Not sufficient parameter alone for successful implantation
Cryopreservation	Vitrification of embryos or gametes	Potential cell damage due to crystal formation,^[^ ^22^ ^]^ inconsistent results, costly^[^ ^23^ ^]^
Preimplantation genetic testing (PGT)	Embryo biopsy and sequencing	Invasive, potential for false negative/positive (due to aneuploidy), costly^[^ ^24^ ^]^
Embryo transfer	Manual catheter‐based embryo transfer with ultrasound guidance	Uterine contractility,^[^ ^25^ ^]^ uterine environment and receptivity,^[^ ^26^ ^]^ potential for ectopic pregnancy due to misplacement of the embryo,^[^ ^27^ ^]^

**Figure 1:**
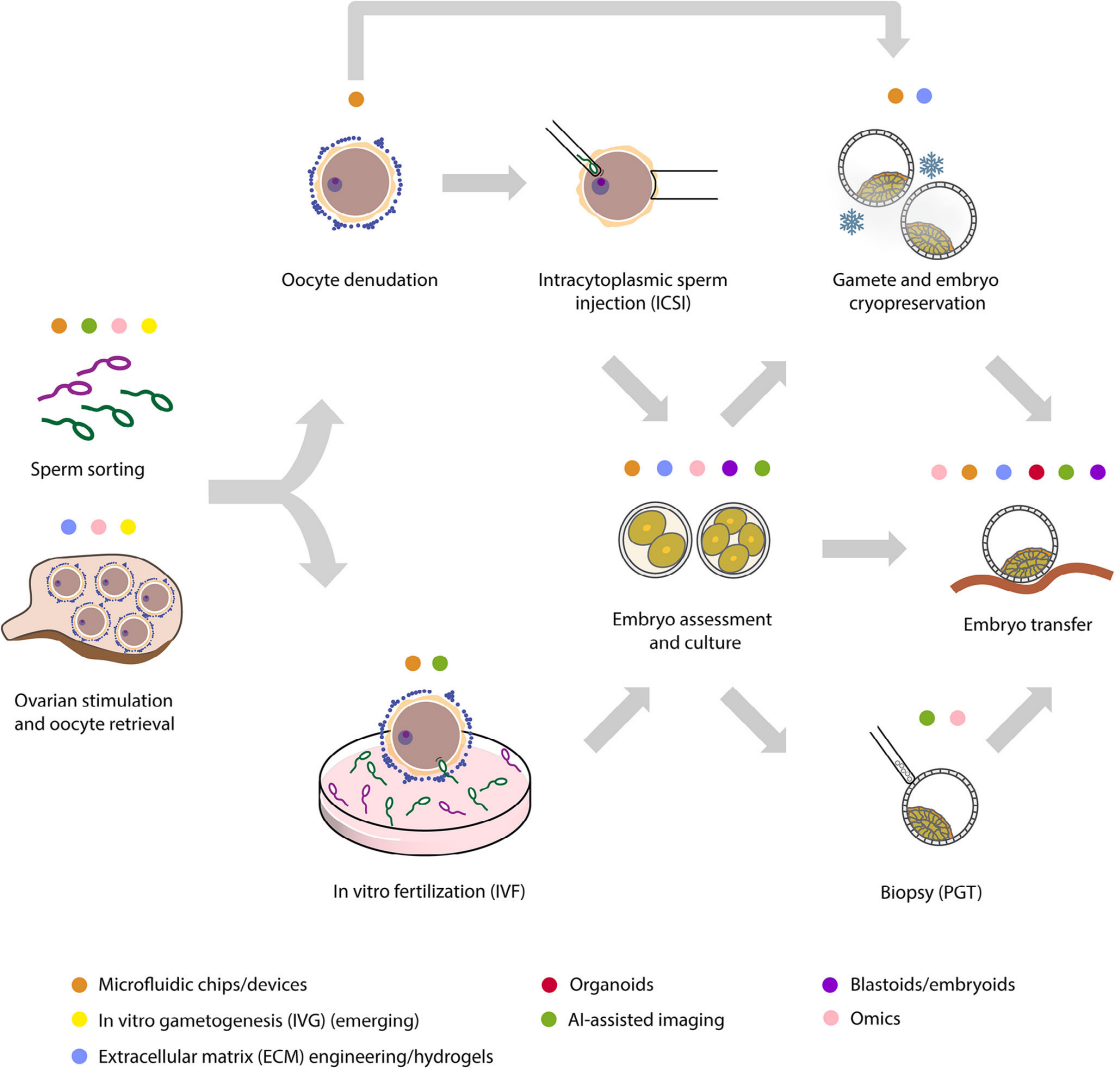
Overview of assisted reproductive technologies (ART) and the integration of emerging bioengineering and computational tools.

As ART continues to evolve, the integration of microengineered tools, stem cell‐based models and computational/bioinformatics tools offer new opportunities to address these limitations (**Figure** **1**). These systems create possibilities to reduce stress on gametes and embryos and automate IVF procedures. This could decrease the cost of ART treatment in the future (reviewed in^[^ ^28^ ^]^), as the number of treatments will be reduced. Additionally, it could reduce the variability in manual processes, typically exposed to sensitivity and subjectivity, while preserving the intrinsic quality of gametes and embryos, which is crucial for successful outcomes.^[^ ^29^ ^]^

The clinical workflow of ART, including both in vitro fertilization (IVF) and intracytoplasmic sperm injection (ICSI), begins with ovarian stimulation, oocyte retrieval, and sperm sorting. In ICSI procedure, initially oocytes are enzymatically or mechanically denuded to expose the zona pellucida. This is followed by cryopreservation of denuded oocytes or direct injection of a single sperm by an embryologist. In IVF, fertilization occurs by co‐incubating selected oocytes with sorted sperm in a culture dish. Following fertilization, both methods proceed with in vitro embryo culture and assessment until the blastocyst stage. At this point, embryos may undergo cryopreservation, biopsy for preimplantation genetic testing (PGT), or be transferred directly into the uterus. Throughout these stages, various bioengineering and advanced biological tools are being applied or developed to improve efficiency, consistency, and predictive power. These approaches are categorized into microfluidic chips/devices, extracellular matrix (ECM) engineering and hydrogels, in vitro gametogenesis (IVG), organoids, AI‐assisted imaging, blastoids/embryoids, and omics technologies. These tools are mapped across ART steps to illustrate their current and potential applications in both clinical and research settings.

### 2.1 Gamete Handling

#### 2.1.1 Sperm Selection and Sorting

Male factor infertility contributes in nearly half of the infertility cases.^[^ ^6^ ^]^ In case of a very low motile sperm count, it is advised to use ICSI for fertilization of oocytes, where single progressive motile sperm cells are selected by the embryologist and injected into an oocyte.^[^ ^30^ ^]^ Sperm selection techniques utilize two main characteristics, density and motility, to identify the highest quality specimens.^[^ ^31^, ^32^ ^]^ Density is used in density gradient centrifugation (DGC), where mature, morphologically normal sperm, being denser, migrate further down a gradient of colloidal silica particles during centrifugation.^[^ ^33^ ^]^ This allows for the separation of higher‐quality sperm from less dense, lower‐quality sperm, and other seminal components. Motility, on the other hand, is at the base of the direct swim‐up (DSU) method, where more motile sperm swim up into an overlay of culture medium, thereby separating themselves from immotile or poorly motile sperm. Both characteristics are important as they correlate with different aspects of sperm quality. Density is associated with sperm maturity and morphology, crucial for successful fertilization, while motility is essential for the sperm's ability to reach and penetrate the egg. However, the traditional selection methods based on centrifugation may lead to damage to the sperm cells, including accumulation of reactive oxygen species (ROS).^[^ ^34^, ^35^ ^]^ Sperm cells are highly sensitive to ROS due to the composition of their membranes, which makes them vulnerable to DNA fragmentation and permanent damage.^[^ ^36^, ^37^ ^]^ Reduced sperm quality can stem from various factors, including loss of chromosomal integrity, elevated ROS levels, and epigenetic changes such as altered DNA methylation, histone tail modifications, and short non‐coding RNAs.^[^ ^38^ ^]^ In ICSI, natural selection processes are bypassed, which could increase the risk of using sperm with fragmented DNA and/or genomic defects.^[^ ^14^ ^]^ Relying solely on morphology and motility may be inadequate for selecting the most viable sperm, which has led researchers to develop alternative methods, including genomics/epigenomics, that consider genetic quality alongside motility. For example, Zhang et al. introduced a novel software tool to assess sperm DNA integrity.^[^ ^39^ ^]^ This non‐invasive, single‐sperm selection approach reduces the subjectivity of manual selection by automating the process and lowering the risk of using sperm with DNA fragmentation in ICSI. Additionally, microengineered platforms could offer the potential to decrease ROS production and preserve DNA integrity.^[^ ^29^ ^]^ In such an attempt, a micro chamber‐based microfluidic platform was used to effectively separate motile sperm from non‐motile sperm and debris in under 10 minutes without sample preparation.^[^ ^40^ ^]^ It significantly improved the mean average‐path velocity and DNA integrity of sperm. Starting with unsorted bovine sample, the platform increased overall sperm motility from 38 to 90% with a 75% retrieval efficiency, making it a high‐throughput, efficient solution for obtaining high‐quality sperm for ART.

**Figure 2:**
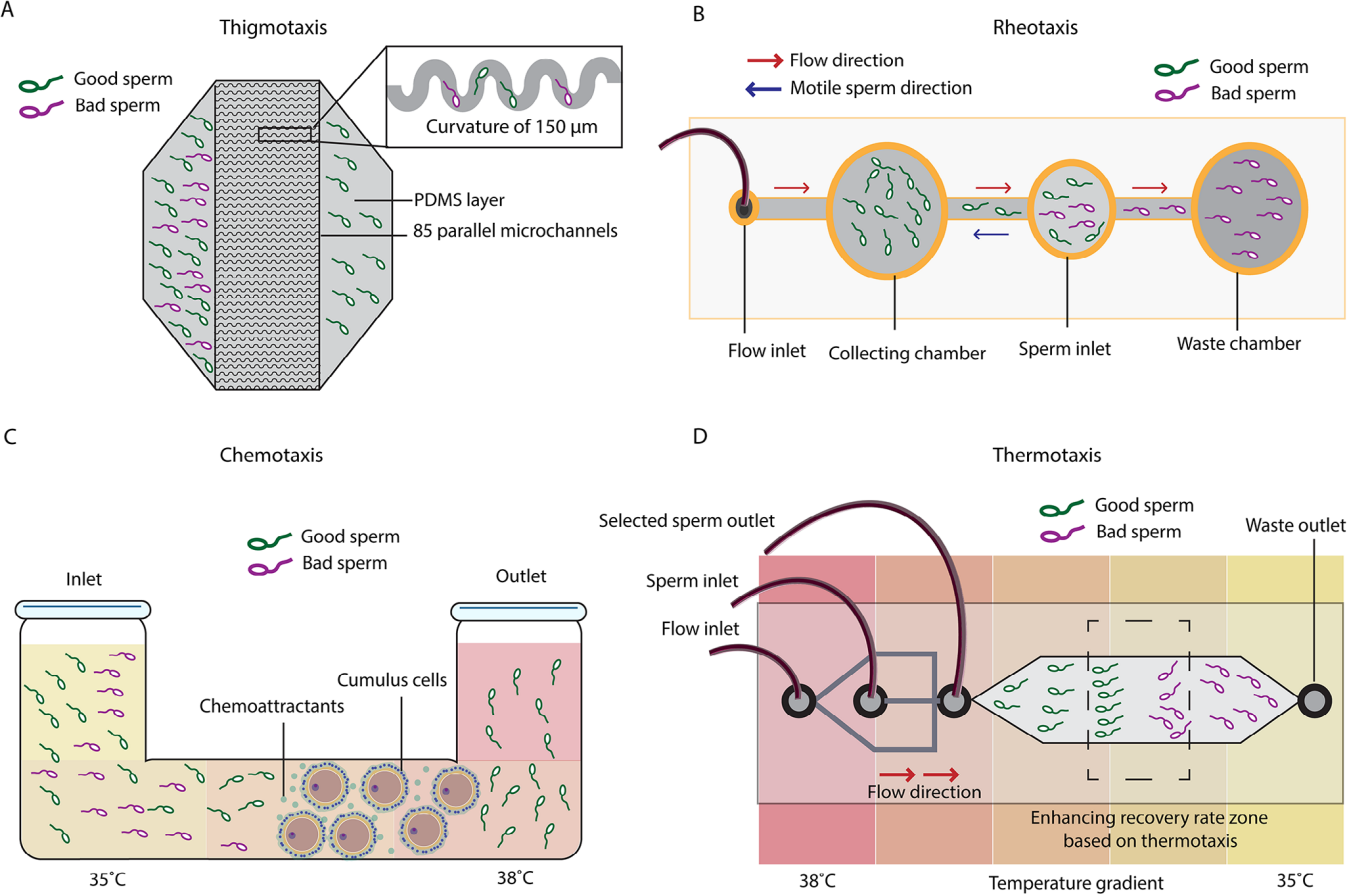
Microengineered platforms for sperm selection based on taxis. A) Microfluidic chip design based on boundary‐following behavior of sperm. This study developed chips with 85 parallel microchannels with 150 µm curvature for selecting good quality sperm. Adapted from.^[^ ^60^ ^]^ B) Microfluidic chip design based on rheotaxis behavior of sperm. This simple setup made use of flow to select good quality sperm based on their ability to swim against. Adapted from.^[^ ^47^ ^]^ C) Microfluidic chip design based on chemotaxis (and thermotaxis). In this setup, cumulus cells of oocytes were placed in the tube for releasing chemoattractant molecules and guiding sperm towards themselves. Adapted from.^[^ ^58^ ^]^ D) Microfluidic chip design based on thermotaxis (and rheotaxis). In this setup, a temperature gradient was applied for creating an enhancing recovery rate zone. The good quality sperm moved toward a more ideal temperature in the gradient, which was further sorted with flow. Adapted from.^[^ ^51^ ^]^

Another advantage of microengineered platforms is the possibility of selecting good quality sperm with higher innate behavioral responses, known as taxis. These include rheotaxis, to swim towards a current;^[^ ^41^, ^42^, ^43^, ^44^, ^45^, ^46^, ^47^, ^48^, ^49^, ^50^, ^51^, ^52^ ^]^ thigmotaxis, tendency to swim along walls;^[^ ^48^, ^50^, ^53^, ^54^, ^55^ ^]^ chemotaxis, to swim towards chemical stimuli, chemoattractant, released by the oocyte;^[^ ^56^, ^57^, ^58^ ^]^ and thermotaxis, to swim towards optimal temperature zones.^[^ ^51^, ^57^, ^58^ ^]^ By mimicking the microenvironment within the fallopian tube, these platforms allow for the efficient and selective sorting of sperm based on their ability to respond to certain stimuli and of better genetic quality. A study by Simchi et al. introduced a sperm selection device that mimics the boundary‐following navigation mechanism, thigmotaxis, found in the female reproductive tract.^[^ ^59^ ^]^ A 3D network of 6500 channels guides viable sperm towards a collection site, isolating high‐quality sperm with high DNA integrity. The device achieved high selectivity and yield, significantly outperforming conventional methods, reducing DNA fragmentation, and streamlining clinical workflow by simplifying the preparation process. Combining rheotaxis and thigmotaxis, Dadkhah et al. developed a cervix‐inspired microfluidic platform (**Figure** **2**A).^[^ ^60^ ^]^ The design of a parallelized chip consisting of 85 microchannels was inspired by the unique serpentine geometry and the sperm's tendency to follow boundaries, which enhanced the selection performance when combined with fluid backflow. This device was able to process 0.5 mL of unsorted semen within 20 min, outperforming the traditional methods in terms of genetic quality. Another example involves a recently developed chip designed to enhance sperm sorting by leveraging the principle of rheotaxis (Figure 2B).^[^ ^47^ ^]^ By introducing shear stress to the semen through a microfluidic flow, the sperm are prompted to swim against the current. This technique results in the collection of only the most motile sperm in the designated chamber. When comparing DNA fragmentation and motility between the flow and static conditions, the flow condition demonstrated lower DNA fragmentation, higher motility, and improved morphology. In another study, combining chemotaxis and thermotaxis for sperm selection was compared to DSU (used as control) (Figure 2C).^[^ ^58^ ^]^ Cumulus cells (CCs) from oocyte were placed in the setup as a potent chemoattractant for sperm. These cells are known to secrete many molecules, including progesterone, which in turn induce various molecular mechanisms in sperm.^[^ ^61^ ^]^ The group showed that spermatozoa capable of passing through CCs exhibited significantly less DNA fragmentation compared to those in control groups. These findings indicated that the chromatin quality of spermatozoa was markedly preserved by passing through CCs. Finally, a study by Huang et al. developed a microfluidic device that incorporated thermotaxis as a key component for sperm selection (Figure 2D).^[^ ^51^ ^]^ Sperm cells are known to be sensitive to temperature changes, and they tend to swim towards slightly warmer temperatures. By utilizing this thermotactic behavior, the microfluidic device encourages sperm to migrate towards the warmer end of the channel. This process helped select sperm with better motility and potentially higher quality, as those capable of responding to the temperature gradient were more likely to reach the collection area.

While most microengineered platforms for sperm selection are based on various forms of taxis, external principles and mechanisms can be utilized for sperm selection. For example, there are studies making use of optical tweezers to manipulate individual sperm cells, allowing precise selection of the healthier sperm based on morphology.^[^ ^62^ ^]^ Additionally, magnetic‐activated cell sorting (MACS) utilizes magnetic nanoparticles conjugated with antibodies that bind to apoptotic sperm. A magnetic field is then applied to remove these labeled sperm, enriching the sample with healthy sperm.^[^ ^63^ ^]^ Alternatively to improving semen selection, the group of Gai et al. used the principle of surface acoustic waves (SAW) on an acoustofluidic platform to boost sperm motility.^[^ ^64^ ^]^ This device consists of a SAW microchip and a polydimethylsiloxane (PDMS) chamber for seeding semen. High frequency ultrasound exposure (≈19 MHz, 2 Watts, and 20 sec) increased the intracellular rate of metabolic activity and thereby increased semen velocity up to 34% and the number of motile sperm cells by 33%. Studies as such are promising for the future of integration of microfluidics into ART procedures (as reviewed in^[^ ^29^ ^]^).

Finally, one needs to ask whether microfluidic‐based semen sorting affects the overall fertility rate. A randomized controlled study was conducted in 2019, where two methods of sperm selection were compared for patients undergoing ICSI in order to assess the male gamete contribution to aneuploidy.^[^ ^65^ ^]^ The control group underwent traditional swim‐up, and the experimental group used a microfluidic ‘Fertile Chip’. This microfluidic device has a membrane filter with pore sizes that allow sperm cell selection.^[^ ^66^ ^]^ This study compared several parameters, including fertilization and implantation rates, and clinical pregnancy rates. Although there was no statistically significant difference between fertilization rates, the fertile chip resulted in higher clinical pregnancy rates (59.4 vs 35.9%, *p* = 0.006). In a more recent, double‐blinded randomized controlled trial, ZyMōt Multi (850 µL) Sperm Separation Device was compared with density gradient centrifugation. It was shown that post‐processing concentration and motility was significantly higher in the microfluidic chip.^[^ ^67^ ^]^ Overall, microfluidic‐based semen selection may improve pregnancy rates and live birth rates in IVF, and particularly ICSI approaches, while at the same time reducing costs and labor time. Thus, these platforms have the potential of becoming a standard tool to improve ART quality.

#### 2.1.2 Removal of Cumulus Cells and Oocyte Cryopreservation

The success of ART heavily relies on efficient oocyte handling, yet two critical steps, cumulus cell removal and cryopreservation, remain significant limiting factors. Traditional methods for cumulus cell removal and oocyte cryopreservation may compromise the viability and developmental potential of oocytes, posing challenges to achieve optimal outcomes. Recent advancements in microengineered platforms present promising alternatives to these conventional clinical methods. By providing precise control over volume, timing and efficiency of these processes, microengineered platforms have the potential to improve oocyte handling in ART, thereby improving both the safety and success rates of fertility treatments.

Factor 1: Removal of cumulus cells. Cumulus cells (CCs) are integral for oocyte maturation, which are densely packed granulosa cells surrounding the oocytes to protect them from the microenvironment.^[^ ^68^, ^69^ ^]^ Although oocyte maturation occurs in a cyclical manner, some patients with fertility issues such as polycystic ovary syndrome or ovarian hyperstimulation syndrome need ART to regulate oocyte maturation.^[^ ^70^, ^71^ ^]^ This is referred to as in vitro maturation (IVM) of oocytes in which the tertiary or antral stage follicles are cultured into MII‐stage oocytes.^[^ ^72^, ^73^, ^74^ ^]^ However, the maturation rates are still relatively low, which may indicate suboptimal culture conditions.^[^ ^75^ ^]^

**Figure 3:**
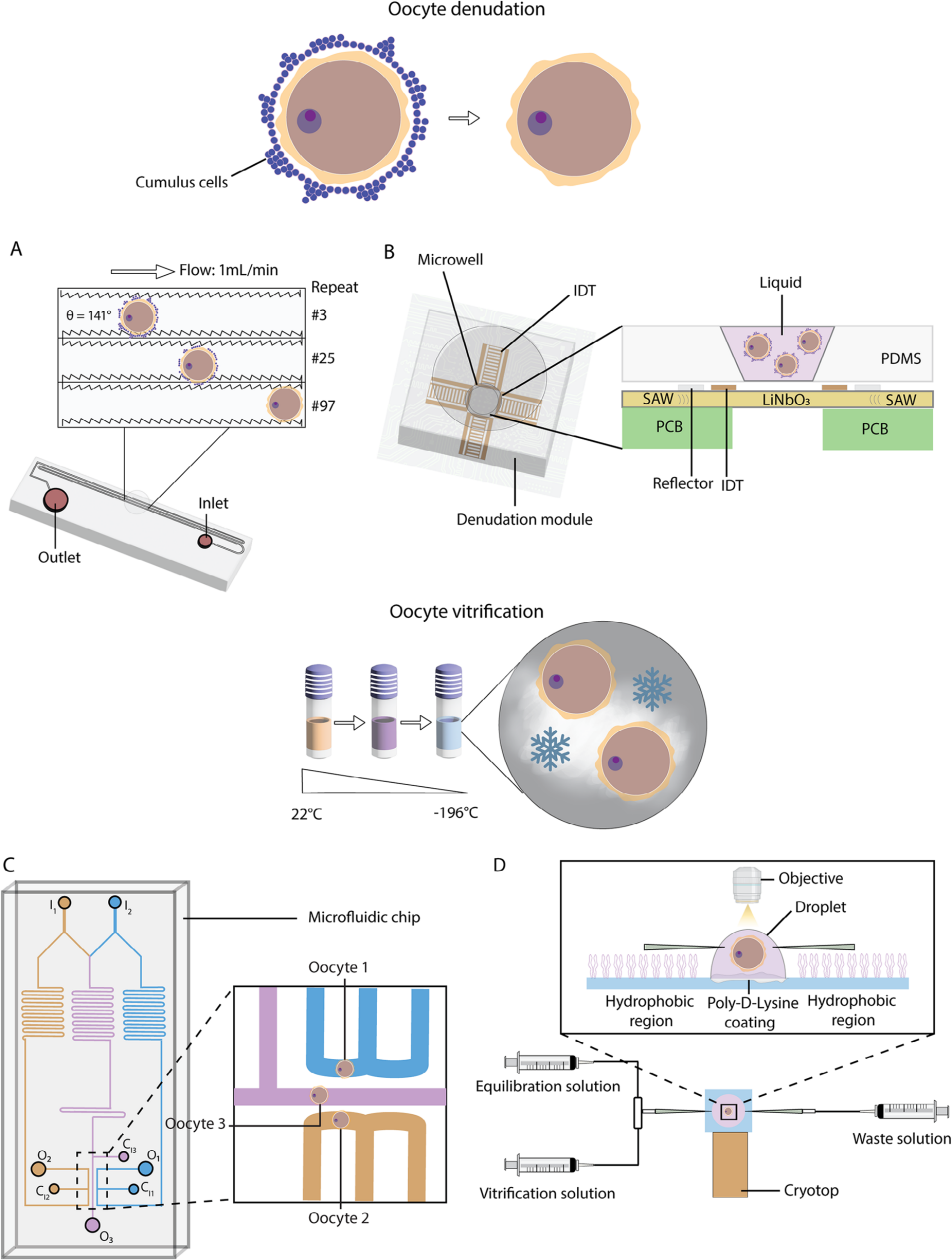
Microfluidic approaches for oocyte denudation from cumulus‐oocyte complexes and for precise oocyte vitrification. A) An oocyte denudation device consisting of 150 constriction‐expansion units with varying geometry (width) and surface (smooth or jagged‐teeth). Tubing attached to syringe pump was pre‐filled with culture medium and was inserted in the inlet to create flow. In the insert, denudation of an oocyte was depicted following the repeated units. Naked oocytes were collected from the outlet. Adapted from.^[^ ^86^ ^]^ B) Oocyte denudation module consisting of microwell and IDTs for generating SAW. The PDMS module was placed on top of printed circuit board (PCB). Side view showing the arrangement of IDTs around the edge of the microwell on top of the LiNbO3 substrate and how the traveling SAW interacts with the liquid and oocytes inside the well. Adapted from.^[^ ^87^ ^]^ C) Microfluidic perfusion chip for studying permeability of multiple oocytes at the same time. A serpentine channel was designed to precisely mix the solution and create varying concentrations, which was used to study how permeability depends on concentration. I_1_ = inlet 1, I_2_ = inlet 2, C_i1_ = cell inlet 1, C_i2_ = cell inlet 2, C_i3_ = cell inlet 3, O_1_ = outlet 1, O_2_ = outlet 2, O_3_ = outlet 3. Magnified view showing three oocytes in different channels with different CPA concentrations. Adapted from.^[^ ^88^ ^]^ D) Microfluidic chip for efficient loading of cryoprotectants for vitrification. The chip was attached to the cryotop (a film strip for vitrification medium volume minimization) and to three different syringes for loading both equilibration and vitrification solutions through the micropipettes on both side and one for waste. The chip consisted of hydrophobic, and poly‐D‐Lysine coated surfaces for stabilizing the droplet containing oocyte. Adapted from.^[^ ^89^ ^]^

For ICSI procedures, CCs need to be removed with a process called denudation, which allows the embryologist to visualize the presence of the second polar body.^[^ ^76^, ^77^ ^]^ Generally, hyaluronidase is used to remove the CCs, which dissolves the hyaluronic acid in the extracellular matrix (ECM) holding the cumulus cells together, allowing them to be detached from the oocyte.^[^ ^78^, ^79^ ^]^ However, prolonged exposure to hyaluronidase can decrease fertilization and developmental ability of oocytes.^[^ ^15^ ^]^ Further, this interaction could lead to the accumulation of by‐products, which could harm the zona pellucida and affect the embryo viability.^[^ ^80^, ^81^ ^]^ Microengineered platforms may be of value for effectively removing CCs from oocytes, without compromising the viability. There have been few attempts in the past to produce such platforms.^[^ ^82^, ^83^, ^84^ ^]^ Zeringue et al. developed a pioneer microfluidic device for cumulus cell removal.^[^ ^85^ ^]^ The group compared the embryo development rate with vortexing and showed that the microfluidic‐based approach led to higher blastocyst formation rates (33 ± 1 to 57 ± 5%, *p* < 0.01). However, this model had several drawbacks, including being able to process only one oocyte at a time and requiring a manual supervision of the flow and switching the flow direction. To overcome these, another group in 2018 developed a PDMS microfluidic device to automate denudation and help reduce cost and time. (**Figure** **3**A).^[^ ^86^ ^]^ The chip had an intriguing design, which consisted of three types of channels, including a repeating jagged‐surface constriction channel. The surface within this channel tilts against (θ = 141°) the flow (1 mL min^−1^) direction to create more friction while the murine oocyte moving along. It was shown that the mean fertilization and blastocysts rates were comparable to the manual denudation methods. In another study, researchers developed a microfluidic chip that made use of ultrasonic SAW generated by four interdigitated transducers (IDTs) arranged symmetrically in a PDMS microwell design (Figure 3B).^[^ ^87^ ^]^ These non‐toxic ultrasonic waves, akin to those used in gynecology and obstetrics, facilitated the denudation of cumulus‐oocyte complexes (COCs) through induced acoustic streaming and radiation force. The device significantly reduced denudation time and labor while maintaining oocyte integrity without loss. Evaluation demonstrated that SAW did not harm oocytes, with comparable post‐ICSI survival, fertilization, and embryo development rates across different groups (80 , 200 MHz, and mechanical pipetting).

Factor 2: Cryopreservation. Oocyte cryopreservation is a highly beneficial method for giving patients the opportunity to have supernumerary embryos from their fresh cycle. It offers fertility preservation to patients who prefer delaying the pregnancy, having ovarian‐associated diseases, or are cancer survivors. Cell recovery is essential when dealing with gametes and embryo cryopreservation, as well as the main obstacle. Even state‐of‐the‐art oocyte cryopreservation methods lead to reduced insemination, showing no sign of fertilization, and blastulation rates compared to fresh oocytes (14.5 ± 9.2 vs 8 ±1.9; 65 vs 54%, *p* < 0.0001, respectively).^[^ ^90^ ^]^ Currently, two oocyte cryopreservation methods are used, namely slow freezing and vitrification (i.e., rapid cooling without ice crystal formation).^[^ ^22^ ^]^ Vitrification requires exposure to higher concentrations of cryoprotective additives (CPA), and therefore, it is currently the method of choice by embryologists.^[^ ^91^, ^92^ ^]^ However, various protocols for vitrification exist, which leads to a variability in outcome and complicates reproducibility.^[^ ^23^ ^]^ These variables include but are not limited to varying CPA concentrations, CPA gradient profiles, temperature profiles and the optimal time of CPA exposure to prevent toxicity,^[^ ^93^ ^]^ which could be reduced with the advantage of microfluidic volumes. Introducing precise and gradual volume of CPAs, as well as different combinations may help with the shock, large osmolality changes and toxicity associated with manual process. Thus, to provide an alternative to manual processing, standardized and automated platforms are developed. One study focused on optimizing the CPA protocol to decrease the osmotic damage, while measuring membrane permeability parameters that could play a vital role in cryopreservation (Figure 3C).^[^ ^88^ ^]^ The chip simultaneously measured three oocytes under the same osmotic shift, enhancing measurement efficiency. A neural network algorithm improved data processing accuracy. Additionally, embryo transfer (ET) showed the microfluidic approach did not harm downstream oocyte development. These features enabled nondestructive, efficient measurement of oocyte permeability, and CPA concentration dependence. Another study, reported in 2021, demonstrated an automated microfluidic‐based vitrification approach to reduce the osmotic shock associated with CPA exposure (Figure 3D).^[^ ^89^ ^]^ The planar microfluidic device consisted of two (super)‐hydrophobic surfaces and in between a poly‐D‐lysine (PDL) coated area for the oocyte containing droplet. This coating allowed the stabilization of the droplet to inject vitrification solutions through micro‐pipettes of the setup. Later, the oocyte shrinkage that takes place during manual vitrification was measured, and it was observed that the oocytes on the microfluidic device (settings: 20 µL, 120 sec, and 1.2 mm) showed 6% reduction in the maximum shrinkage and showed improved survival rate due to reduced impact of an osmotic shock. Alternatively, hydrogel‐based microencapsulation, such as embedding cells in alginate,^[^ ^94^, ^95^, ^96^ ^]^ offers a strategy to minimize extracellular ice‐induced damage by forming a semi‐permeable protective shell around the cells. This encapsulation can regulate the diffusion of CPAs, thereby reducing osmotic shock and improving post‐thaw cell viability.^[^ ^97^ ^]^

In summary, while microengineered platforms show promise for enhancing specific aspects of ART, such as oocyte denudation and cryopreservation, their current application does not yet demonstrate a clear advantage over traditional practices used in IVF laboratories. However, the use of advanced biological models could offer valuable insights to overcome existing limitations. For instance, in vitro gametogenesis (IVG)‐derived oocytes may serve as a robust platform for optimizing denudation and cryopreservation techniques under controlled conditions. These models could help refine protocols and identify critical parameters that are challenging to assess with clinical samples. Nevertheless, significant gaps remain in our understanding of oocyte biology, which limits the full potential of these approaches. The scarcity and precious nature of oocytes, particularly in clinical settings, makes it difficult to obtain sufficient material for research and to induce collaborations between clinicians and scientists.

#### 2.1.3 In Vitro Gametogenesis and Modeling of Gametes

In vitro gametogenesis (IVG) is an experimental procedure bridging reproductive medicine and stem cell fields, which aims to generate gametes in vitro. It is under debate whether it could be considered as a tool for ART, as currently it is highly experimental.^[^ ^98^ ^]^ First, it has, in theory, the potential of making it possible for LGBTQ+ or single parents to procreate, without any other genetic contributions.^[^ ^98^ ^]^ Second, it could provide long‐term solutions for female patients, including pediatric cancer patients, women who suffer from early menopause and infertility.^[^ ^99^, ^100^, ^101^ ^]^ In addition, understanding the biology behind germ cells could lead to understanding and treatment of infertility. Two techniques exist to create in vitro‐derived gametes, using gonadal stem cells (GSCs) and pluripotent stem cells (PSCs), the latter including embryonic stem cells (ESCs) and induced pluripotent stem cells (iPSCs).^[^ ^102^ ^]^ Both techniques involve a two‐step method, where the main cell source is differentiated into primordial germ cell‐like cells (PGCLCs), and then PGCLCs are induced to form gamete cells. Primordial germ cells (PGCs) are the precursors of gametes, which follow certain biological steps. Starting with the development and formation of the germ layers, followed by specialization into male or female germ cells and finally, gametogenesis.^[^ ^103^ ^]^ Complex molecular mechanisms cannot be precisely and in real‐time studied in vivo. Moreover, our knowledge on gamete development, differentiation and maturation is mostly based on mice models.^[^ ^104^ ^]^ It has become evident that human mechanisms are different from those in mice.^[^ ^105^, ^106^ ^]^ For example, in human PGCs, SOX17 is a key transcription factor and promotes mesoderm development, whereas in mouse PGCs, SOX17 appeared dispensable.^[^ ^107^, ^108^ ^]^

Keeping this in mind, researchers have demonstrated differentiation into early mesoderm‐like cells (iMeLCs) from human iPSCs (hiPSCs), which effectively are generated from human PGCLCs with robust capacity for both spermatogenesis and oogenesis.^[^ ^109^ ^]^ This work confirmed that SOX17 is a crucial regulator for PGCLC differentiation. In a later study, using ESCs and iPSCs, researchers have demonstrated in vitro differentiation into advanced male germ cell lineages, including spermatid‐like cells, without genetic manipulation.^[^ ^110^ ^]^ This study effectively mirrored in vivo spermatogenesis, producing cells with key markers and genomic imprints similar to human sperm, offering a novel approach to studying spermatogenesis in vitro. Taking it to the next step, Hwang et al. published their work on PGCLCs differentiation into pro‐spermatogonia by using hiPSCs.^[^ ^111^ ^]^ They modified a xenogeneic method that was developed for pre‐meiotic oogonia‐like cells to their human PGCLCs to induce differentiation towards T1 phase, which is the primary transitional pro‐spermatogonia.

On the oocyte side, in 2017, Jung et al. differentiated human ESCs into ovarian follicle‐like cells (FLCs) in vitro.^[^ ^112^ ^]^ The resulting FLCs resembled primordial follicles, as confirmed by transcriptome analysis, immunostaining, and transplantation experiments. Moving further, to maintain follicle‐oocyte interaction, Laronda et al. developed a bioprosthetic ovary using murine follicles, creating microporous gelatin scaffolds via extrusion‐based 3D printing.^[^ ^113^ ^]^ This study paved the way for the establishment of human folliculogenesis, which in the end could be a solution for patients with ovarian dysfunction. Another study showed the effect of ECM composition on oogonial stem cells (OSCs) differentiation from in vitro derived oocytes.^[^ ^114^ ^]^ It was shown that the contact of mouse OSCs with collagen type‐IV and ‐I induced differentiation and oogenesis with the addition of 500 µm L^−1^ of Arg‐Gly‐Asp (RGD) cross‐linking peptide.

Collectively, bioengineered tools will allow scientists to study the complex communication during fertilization and development in vitro, and shed light onto infertility‐related pathologies tied with these mechanisms. Incorporating microfluidic systems for PGCLCs differentiation from iPSCs may be the next step, as seen with other cell types.^[^ ^115^, ^116^ ^]^ Specifically, these systems could optimize the differentiation and maturation processes. In vivo, germ cell differentiation relies heavily on interactions with supporting somatic cells, such as Sertoli cells in the testes or granulosa cells in the ovaries.^[^ ^117^, ^118^, ^119^ ^]^ Microfluidic devices can create controlled co‐culture zones that mimic these in vivo niches. By positioning PGCLCs near supporting cells (e.g., Sertoli cells), essential paracrine signaling and physical interactions critical for germ cell maturation could be enabled.^[^ ^120^ ^]^ Moreover, embedding gradient‐generating features within microfluidic channels could deliver precise concentrations of key morphogens, such as BMPs,^[^ ^121^, ^122^, ^123^ ^]^ WNTs,^[^ ^124^ ^]^ and retinoic acid,^[^ ^125^ ^]^ at optimal time points, enhancing the differentiation process. The physical properties of the cellular microenvironment, such as stiffness and elasticity, also play significant roles in germ cell development.^[^ ^126^ ^]^ It was recently shown that the matrisome landscape for germ cell development requires constant change.^[^ ^127^ ^]^ Using a dynamic hydrogel matrix that could support the remodeling throughout differentiation or could reflect the natural stiffness of reproductive tissue could support PGCLC differentiation. In another recent study, oocyte maturation from secondary to antral follicles was successfully demonstrated using alginate‐reconstituted basement membrane encapsulation.^[^ ^128^ ^]^ Once the methodology was established, the researchers transplanted oocytes from young mice into aged follicles to evaluate the effects of somatic cell aging on oocyte quality, with oocytes transplanted into young follicles serving as the control. The results revealed that aged follicles impaired meiotic maturation, as evidenced by chromosome misalignment and aberrant spindle formation. In contrast, transplanting aged oocytes into young follicles reduced cell death rates and improved maturation outcomes. This study underscores the critical role of the ECM in oocyte maturation, likely through signaling cascades influenced by the aging process. Finally, germ cell differentiation is a dynamic process sensitive to environmental conditions, including oxygen levels, pH, and nutrient availability. While static cultures often lack this level of control, microengineered platforms might offer continuous monitoring combined with artificial intelligence (AI)‐based image analyses and fine‐tuning of these environmental factors, creating a more physiologically relevant environment that could further optimize germ cell maturation. Overall, IVG offers the potential to produce quality and uniform gametes and enables the generation of patient‐specific sperm and eggs, thereby contributing to ART success rates.

### 2.2 Embryo Handling and Culture

During IVF, the embryo is cultured in vitro until day 3–5 before being transferred to the uterus, containing 8 to 60 cells. Therefore, culture conditions are of utmost importance. As the epigenome of the embryo undergoes drastic changes during the preimplantation development window, the embryo is highly vulnerable. The Developmental Origins of Health and Disease (DOHaD) hypothesis suggests that early life exposures, including the preimplantation period, can influence long‐term health outcomes.^[^ ^129^ ^]^ While there is evidence supporting the potential for IVF culture conditions to affect birthweight and metabolic phenotypes, the long‐term consequences for diseases such as type II diabetes and cardiovascular conditions in humans are still being studied.^[^ ^130^, ^131^, ^132^ ^]^ It has been shown that the success of ART is greatly affected by the dynamic micro‐ and macro‐environment in which the embryo is cultured, as well as chemical and physical factors.^[^ ^133^ ^]^ Chemical factors include water, amino acids, energy substances (pyruvate, lactate, glucose), and vitamins,^[^ ^134^, ^135^, ^136^, ^137^, ^138^ ^]^ while the physical factors include temperature, oxygen tension, humidity and shear stress.^[^ ^139^, ^140^, ^141^ ^]^ Glucose and amino acids are particularly required for the developing embryo, since these substances trigger rapamycin (mTOR) pathways.^[^ ^142^ ^]^ These pathways are crucial for growth and development of the embryo. Despite exhaustive studies aimed at optimizing culture systems,^[^ ^62^, ^143^, ^144^, ^145^, ^146^, ^147^, ^148^, ^149^ ^]^ several challenges persist. For example, during IVF, supernumerary embryos are formed, some of which may undergo preimplantation genetic testing (PGT) to detect abnormalities.^[^ ^150^ ^]^ Excess embryos are often cryopreserved for future use, adding to the financial burden of IVF cycles.^[^ ^151^ ^]^ Moreover, it is common practice to use oil in micro‐drops to prevent evaporation and absorption of toxic substances during static in vitro culture of embryos.^[^ ^152^ ^]^ However, it was shown that the mineral oil absorbs lipophilic factors, which may lead to the entrance of damaging molecules into the embryo medium and affect embryo outcome.^[^ ^21^ ^]^ Finally, the pipetting process could lead to increased shear stress that in turn has adverse effects on embryo cells.^[^ ^153^ ^]^ In vitro manipulations during embryo culture and transfer have been implicated to cause low pregnancy rates, contributing to variations in success rates among different IVF laboratories.^[^ ^19^, ^20^ ^]^ It was shown that shear stress is sensed by the cells and at 2 dynes cm^−2^, leads to induction of MAPK8/9 phosphorylation and therefore to cell death after 12 h.^[^ ^154^ ^]^ When considering all factors, culture conditions differ across laboratories, encompassing variations in the number of cultured embryos (either in groups or individually), medium volume, the application of mineral oil, and the stimuli experienced by embryos, as thoroughly reviewed in Le Gac et al.^[^ ^155^ ^]^ Finally, embryo aneuploidy is a major limiting factor in ART success. It is defined as the presence of (partial) abnormal numbers of chromosomes in cells, resulting in effected chromosomal segment.^[^ ^156^ ^]^ Aneuploidy is a significant cause of infertility, accounting for 10–30% of miscarriages.^[^ ^157^, ^158^ ^]^ Chromosomal abnormalities can arise from meiotic errors, resulting in uniform abnormalities across all gamete cells, or from mitotic errors, leading to mosaicism, a condition where different cell populations exhibit varying chromosomal abnormalities. While earlier reports indicated that 50% of embryos created for IVF were diagnosed with mosaicism involving numerical and structural abnormalities,^[^ ^159^ ^]^ a recent study using single‐cell DNA sequencing suggests this rate is actually over 80%^[^ ^160^ ^]^ and possibly even higher. Finally, maternal age is a crucial factor, as embryos produced by women aged over 37 may exhibit developmental arrest or implantation failure.^[^ ^161^, ^162^ ^]^

**Figure 4:**
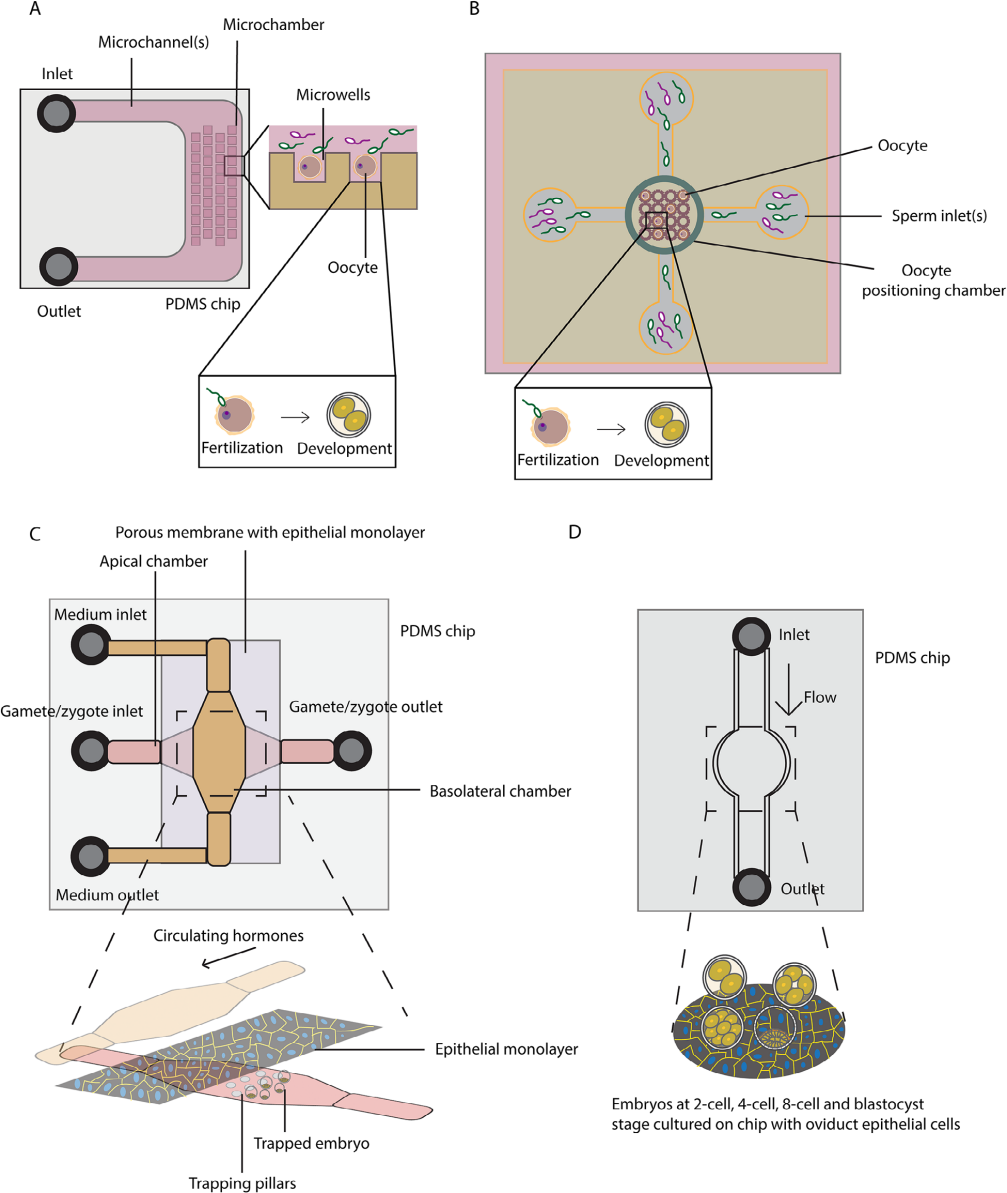
Microfluidic platforms for in‐chip fertilization and blastocyst culturing as an alternative to static culture in IVF labs. A) Microfluidic design for in vitro fertilization. This chip had an integrated oocyte‐positioning system for motile sperm selection and fertilization. Switching from fertilization medium to development medium allowed embryos to develop into blastocyst stage. Adapted from.^[^ ^163^ ^]^ B) A simpler microfluidic design for oocyte trapping. In this setup, the micro‐chamber consisted of microwells allowed the oocyte trapping for fertilization. Flow was introduced for medium substitution and debris cleaning. Adapted from.^[^ ^164^ ^]^ C) Microfluidic chip designed for trapping gamete/zygotes by the trapping pillars on the apical chamber. The porous membrane above the apical chamber seeded with oviduct epithelial monolayer provided molecules for embryo development. On the top, the basolateral chamber provided flow which led to hormone circulation. Adapted from.^[^ ^167^ ^]^ D) A simpler microfluidic chip design for embryo culture. Oviduct epithelial cells were seeded in the chamber prior to embryo culture. Tubes were attached for automated medium changing. Embryos from different stages were cultured on the chip, with and without the epithelial cells. Adapted from.^[^ ^168^ ^]^

Before embryo development, successful fertilization needs to take place. The development of IVF‐on‐a‐chip marks a significant advancement in reproductive technology, providing a controlled and extendable miniaturized environment for more sophisticated embryo culture and monitoring techniques. One of the earliest attempts for developing such a platform is from Han et al., where the group designed a microwell structured for oocyte trapping for fertilization (**Figure** **4**A).^[^ ^163^ ^]^ Microwells both served as trapping of the oocytes, as well as tracking of single embryos. The embryos were successfully cultured until the blastocyst stage. Advancing the platform, Ma et al. developed a fully integrated IVF system, which included sperm screening, fertilization, embryo culture and embryo screening (Figure 4B).^[^ ^164^ ^]^ Using their oocyte positioning chamber in the middle for oocyte trapping, and channels for sperm screening, the group achieved fertilization with an embryo growth rate comparable to traditional IVF protocol. Although these platforms have not yet demonstrated better outcomes compared to traditional protocols, they offer dynamic systems with potentially higher physiological relevance to in vivo conditions, making them promising candidates for becoming the gold standard. Their microfluidic design enables efficient nutrient and gas exchange, minimizes the use of expensive media, and reduces the risk of polyspermy.^[^ ^17^ ^]^ Additionally, these advanced platforms could integrate additional cell types, such as oviductal^[^ ^165^ ^]^ and endometrial cells, creating a sequential culture environment that supports embryo development more naturally. Unlike traditional methods that rely on static conditions, mineral oil overlays, extensive pipetting, and large volumes, microfluidic systems provide a controlled, fluidic environment that enhances nutrient and gas exchange. These dynamic culture systems may reduce mechanical stress on embryos, allow standardization of an automatic workflow, and require significantly smaller volumes, thereby improving overall efficiency and potentially increasing the success rates of IVF procedures. In such an attempt, Mancini et al. used a microfluidic device to culture murine blastocysts in a controlled environment combined with endometrium cells.^[^ ^166^ ^]^ The group used the device to carry out metabolomics analysis by reverse phase liquid chromatography and untargeted positive ion mode mass spectrometry. It was shown that the blastocyst rate was significantly higher when co‐cultured with endometrium cells compared to control condition in the microfluidic device (71.8 ± 4.3 vs 54.6 ± 6.6%). In addition, the device could be used effectively to delineate the amino acids necessary for embryo development, such as arginine, proline, and pyrimidine.

The optimal approach for culturing embryos involves replicating the microenvironment as closely as possible, with the oviduct serving as a key reference point, as extensively reviewed.^[^ ^169^ ^]^ The brief residence of embryos in the oviduct allows for crucial developmental processes, facilitated by the provision of ECM, essential nutrients, light exposure, optimal temperature, and oxygen tension.^[^ ^170^, ^171^, ^172^, ^173^ ^]^ Recognizing this, a research group in 2018 developed a PDMS oviduct‐on‐chip model composed of oviductal epithelial cells (Figure 4C).^[^ ^167^ ^]^ This system was tested using bovine zygotes, and comparisons of epigenetic modifications such as methylation with in vivo zygotes revealed notable similarities. Despite demonstrating some success in mimicking epigenetic changes, limitations persist, particularly in achieving a sufficient number of zygotes at the 8–16 cell stage and enhancing the microenvironment regulated by ovarian hormones. In addition to the aforementioned research, a more recent study introduced a microfluidic chip designed to mimic the environment of the oviduct (Figure 4D).^[^ ^168^ ^]^ This study compared the levels of ROS production, indicative of embryonic distress, across different experimental conditions, including chips with oviductal cells, chips lacking cells, and static drop culture. The findings revealed decreased ROS production in 4‐cell stage embryos cultured on chips containing oviductal cells, highlighting the potential benefits of this microenvironment model. Increased investment in the development of reliable oviduct‐on‐chip platforms could substantially improve embryo culture conditions.

Looking ahead, introduction of extracellular vesicles (EVs) to in vitro culture of embryos could be beneficial for embryo development. EVs are double‐layered lipid membrane vesicles and due to their enclosed structure, EVs could carry signaling molecules to long distances without being degraded.^[^ ^174^, ^175^, ^176^ ^]^ These structures have been identified in uterine fluids of various species, including humans, sheep and mice.^[^ ^177^, ^178^, ^179^ ^]^ These findings suggest that EVs play a part in the implantation of embryo. A study was performed by Qu et al, where the group cultured white New Zealand rabbit embryos in oviduct fluid supplemented with EVs and melatonin, and showed reduced ROS and 5‐mC levels compared to in vitro control group, as well as increased blastocyst rate.^[^ ^180^ ^]^ Here, culturing EVs with microfluidic systems could improve the culture outcomes, where the microfluidic devices can be designed to efficiently load EVs into the culture medium. This can be achieved through precise manipulation of flow and mixing within the microchannels to ensure uniform distribution of EVs. Additionally, microfluidic devices can facilitate the localized delivery of uterine EVs to embryos, concentrating EVs around developing embryos. This can be achieved, for example, by adapting the oocyte‐trapping platform developed by Ma et al.^[^ ^164^ ^]^ This targeted delivery can enhance the uptake of EVs by the embryos and maximize their biological effects.

A futuristic approach may involve bypassing in vitro culture entirely by delivering the embryo directly to the oviduct or endometrium. This emerging option was recently explored in depth in the literature, particularly in,^[^ ^181^ ^]^ which extensively reviews microrobots categorized as tethered, untethered, or combined. These microrobots hold promise for precisely delivering embryos to their intended sites, facilitating development under natural conditions. For instance, grippers, the most utilized robotic method for cargo delivery, can be adapted using magnetic or thermal modifications. Once the gripper reaches the fallopian tube, the necessary stimuli can be applied to release the embryo. Another advantageous feature of grippers is their ability to be manufactured from various materials, allowing for adaptation to different microenvironments.

## 3 Microengineered and Stem Cell‐Based Tools for Modelling Implantation

Reproduction in humans is highly vulnerable. Various external and internal factors could disrupt the sequence until birth, starting with fertilization. In the case of viable offspring, it does take a village of specific organs, tissues, and cells with well‐orchestrated signaling pathway activity.^[^ ^182^ ^]^ The molecular basis of implantation biology remains yet to be discovered in‐depth, mainly because our understanding of these mechanisms is based heavily on animal studies.^[^ ^183^ ^]^ When translated into clinical practice, implantation, which is the key to a successful pregnancy, remains to be a limiting factor for IVF success and is also called the “black box” of reproduction. Unraveling the morphogenetic and molecular mechanisms that take place naturally during pre‐ and peri‐implantation will help scientists close in on identifying the underlying causes of infertility and potential therapeutic options. In this section, we highlight two advanced stem cell‐based models of the early embryo and endometrium, as the main players of implantation. By providing a controlled environment to study the multitude of interactions between the embryo and maternal tissues, these models may offer valuable insights that can improve ART outcomes, such as increasing implantation rates and addressing implantation failures, ultimately leading to better clinical practices and personalized treatment strategies.

### 3.1 Modeling Pre‐ and Peri‐Implantation Embryo Development using Stem Cells

Egg activation is triggered with the penetrance of sperm. With an “out with the old, in with new” approach, a remarkable transition is observed from a fertilized oocyte to a totipotent zygote.^[^ ^184^ ^]^ This transition is marked by the formation of pronuclei followed by the initiation of the first mitotic division. During embryonic genome activation, the zygote takes over the genetic control while the maternal genome degrades.^[^ ^185^ ^]^ ≈16 cell‐stage, the blastomeres form a compact cluster high in tight junctions, named the morula.^[^ ^186^ ^]^ This event leads to the first specialization of cells in the periphery of the embryo becoming the extraembryonic trophoblast cells. These cells will absorb fluid and form the blastocoel cavity, which expands leading to a blastocyst structure.^[^ ^187^ ^]^ Following the first lineage bifurcation, the pluripotent inner cell mass (ICM) is formed, as well as trophectoderm (TE), which will differentiate into the trophoblast cells that form the placenta.^[^ ^188^ ^]^ Following the second lineage bifurcation, the ICM specializes into epiblast (Epi), which will become the embryo proper, and hypoblast (HYPO), which will give rise to extra‐embryonic endoderm tissues and contribute to the gut.^[^ ^189^, ^190^, ^191^ ^]^ Our knowledge of embryogenesis heavily relies on animal models and is limited by the accessibility to natural embryos and by ethical regulations such as the 14‐day culture limitations^[^ ^192^ ^]^ Recently, mouse^[^ ^193^, ^194^, ^195^ ^]^ and human^[^ ^196^, ^197^, ^198^, ^199^, ^200^, ^201^, ^202^, ^203^, ^204^ ^]^ stem cell‐based models of the blastocyst were developed from both embryonic stem cells (ESCs) and induced pluripotent stem cells (iPSCs) expanded in naïve pluripotent conditions, named blastoids (as reviewed in^[^ ^205^ ^]^). Blastoids offer several advantages over blastocysts, including their generation in large numbers and amenability to genetic engineering. Microwell‐based culture platforms are state‐of‐the‐art platforms for culturing blastoids. Our group has previously generated agarose hydrogel microwell screening platforms using replica molding techniques in order to screen and automate the imaging of the blastoids while keeping them in close contact to each other, as well as homogenizing the formation of the structures.^[^ ^206^ ^]^ In an alternative microwell culture platform, the microwell arrays were fabricated using a microthermoforming process of thin thermoplastic polymer films like cyclic olefin copolymer (COP), which improves confocal imaging readouts.^[^ ^206^, ^207^ ^]^ In 2018, the first blastoid study, mouse ESCs were combined with mouse trophoblast stem cells (TSC) in hydrogel microwells and showed that signals from the epiblast drive trophectoderm development and cavitation.^[^ ^195^ ^]^ Following this pivotal study, scaled‐up experimentation using microwell‐based screening combined with high‐content imaging approaches has been used for identifying novel signaling pathway regulators to improve blastoid formation further.^[^ ^208^, ^209^, ^210^ ^]^

In 2021, multiple independent groups^[^ ^196^, ^197^, ^198^, ^202^ ^]^ showed the formation of human blastoids using microwell array culture platforms. Kagawa et al. showed efficient formation in the hydrogel microwell screening platform by inhibiting the three developmental signaling pathways: Hippo, TGF‐β, and ERK.^[^ ^202^ ^]^ Furthermore, Karvas et al. used the commercial Aggrewell platform to generate blastoids that showed the capacity for extended culture up to 21 days and presented hallmarks of primitive streak and the emergence of embryonic germ layers.^[^ ^211^ ^]^ Overall, blastoids provide a model to study the early stages of human development and the implantation process with reduced ethical concerns than associated with using actual human embryos. This can increase our understanding and potentially identify new therapeutic routes for implantation failures and recurrent miscarriages. Embryo models can also be valuable for studying rare events that are difficult to observe in ART but nonetheless occur and can impact ART outcomes. For instance, monochorionic twinning, in which monozygotic twins share the same placenta, appears to be linked to ART procedures, with incidence rates at least twice as high as in natural conception.^[^ ^212^ ^]^ Monochorionic twins are associated with significantly increased pregnancy‐related risks, including twin–to–twin transfusion syndrome (TTTS) and placental insufficiency, both of which elevate the risk of pregnancy loss.^[^ ^213^, ^214^ ^]^ We recently developed a bioengineered human embryo model for monochorionic twinning.^[^ ^215^ ^]^ Here, screening assays using microwell arrays have proven useful in identifying the culture conditions that allow reproducible formation of high numbers of monochorionic twin blastoids. Specifically, increased cell numbers in combination with strong Hippo signaling inhibition induced the division of the ICM into two, both surrounded by a single trophectoderm cyst. The thin polymer film‐based thermoformed microwells allowed time‐lapse fluorescence imaging using an OCT4 reporter to further explore twinning morphogenesis. This novel model paves the way to deepen insights into the uncovered mechanisms underlying twinning.

Moving towards peri‐ and post‐implantation in human, a distinction can usually be made between integrated and non‐integrated models. Integrated models aim to replicate both the embryo itself and all the supportive tissues essential for embryonic development. These models are designed to mimic the early stages of human embryogenesis, including the formation of extraembryonic structures such as the placenta and yolk sac. Non‐integrated models focus on mimicking only a specific component of the embryo rather than the complete structure. As non‐integrated models do generally not possess trophectoderm/trophoblast cells, these structures cannot form the placenta and are therefore viewed as ethically distinct and in some respects, less morally contentious. With an increasing number being published, and with varying decrease of developmental potential, both integrated and non‐integrated embryo models are valuable tools in biomedical research (**Table** **2**).

**Table 2:** Recent developments of integrated and non‐integrated human embryo models.

Model	Starting cell type	Peri‐ /post‐implantation development	Integrated/non‐integrated	Reference
Human Blastoids	Naïve human PSCs	TE, Epi, and HYPO. Formation of the blastoid cavity	Integrated	[196]
iBlastoids	Human dermal fibroblast	TE‐ and Epi‐like layers with cavitation (HYPO not mentioned)	Integrated	[197]
Human blastoids	Naïve human PSCs	TE, Epi, and HYPO. Emergent yolk sac‐like and amniotic‐like cavities.	Integrated	[198]
EPS‐Blastoids	Extended PSCs	TE, Epi, and HYPO. TE differentiation	Integrated	[199]
hEP‐structures	Expanded PSC	Extra‐embryonic‐like compartment, HYPO‐like specification	Non‐integrated	[200]
Blastoids	human totipotent blastomere‐like cells (hTBLCs)	TE‐ and EPI‐like layers with compaction and polarization	Integrated	[216]
Human Blastoids	Naïve human PSCs	TE, Epi and HYPO. Limited TE differentiation, pro‐amniotic‐like cavities	Integrated	[202]
Blastoids	human 8C‐like cells (8CLCs)	TE‐ and EPI‐like layers with compaction	Integrated	[203]
Blastoids	Intermediate cells of the primed‐to‐naïve transition process	TE, Epi, and HYPO. Pro‐amniotic cavity‐like and primary yolk sac‐like structures	Integrated	[204]
Spontaneous blastoids	hnESCs	TE, Epi, and HYPO. Blastoid mesoderm (bMES), definitive endoderm (bDE), and hemogenic endothelium (bHE)	Integrated	[217]
Twin blastoids	Naïve human PSCs	TE, Epi, and HYPO. Dual ICM development.	Integrated	[215]
heX‐embryoid	iPSCs	Extra‐embryonic endoderm, mesoderm, and epiblast	Non‐integrated	[218]
nHyCs	Naive hPSCs	Bilaminar structure with a pro‐amniotic‐like cavity	Non‐integrated	[219]
hEEs	hPSCs	Amniotic ectoderm, primitive streak, mesoderm, and early extra‐embryonic endoderm	Non‐integrated	[220]
E‐assembloids	Naive hPSCs	Extra‐embryonic endoderm‐like cells	Non‐integrated	[221]
Human gastruloids	hPSCs	Epiblast‐like, Extraembryonic‐like	Non‐integrated	[222]
Synthetic embryo models (SEM)	Naive transgenic hPSCs	TE, Epi, and HYPO. Extra‐embryonic mesoderm.	Integrated	[223]
Human embryoids	Naive hPSCs	TE, Epi and HYPO. Lumenogenesis, amniogenesis, primordial germ cell formation and specification of the anterior hypoblast	Integrated	[224]

For ART research, human blastoids appear to be the most suitable models by their resemblance to the full integrated preimplantation embryo and their ability to be generated in large quantities. While there is room for improvement in human blastoids, such as improving the HYPO compartment, they offer valuable applications. For instance, they could be used to train embryologists in blastocyst handling techniques, providing an ethical and reproducible alternative to human embryos. In addition, under the right guidelines, blastoids and embryo models by‐pass more strict ethical regulation surrounding the use of blastocyst for experimentation, including the 14‐day rule.^[^ ^225^ ^]^ Although the 14‐day rule was originally established to regulate research on fertilized human embryos, stem cell‐derived models such as blastoids occupy a regulatory gray zone due to their non‐embryonic origin and limited developmental potential. In recognition of this, the 2021 guidelines of International Society for Stem Cell Research (ISSCR) reconsidered the strict 14‐day limit, recommending case‐by‐case ethical oversight for extended culture, as in the case of Karvas et al. study.^[^ ^211^ ^]^ Advances in bioengineering may further enable the development and analysis of these models beyond traditional boundaries, necessitating renewed ethical frameworks in parallel. Relating single‐cell and omics studies of embryo models and embryos offers a powerful approach to gaining deeper insight into early development and implantation processes.^[^ ^226^, ^227^, ^228^ ^]^ This integrated strategy could improve in vitro culture conditions that in turn could increase success rates in ART. Typically, plastic substrates or ECM‐like matrices are used for the progressive culture of blastoids and blastocysts towards post‐implantation stages.^[^ ^229^, ^230^ ^]^ However, advanced in vitro models of the endometrium could improve post‐implantation development of blastoids and the spatiotemporal resemblance to natural development, with impact on modelling reproductive health and disorders.

### 3.2 Modeling Implantation using In Vitro Bioengineered Endometrium

Implantation is an intricate yet enigmatic process involving crosstalk between the endometrium and the blastocyst embryo. This critical event unfolds in three steps following blastocyst hatching.^[^ ^231^ ^]^ Implantation commences with the apposition of the blastocyst in close proximity to the luminal epithelial lining of the endometrium.^[^ ^232^ ^]^ This marks the initial contact between the two entities.^[^ ^233^ ^]^ This contact is followed by adhesion, where the trophectoderm at the embryonic pole engages with the luminal epithelium mediate by a myriad of adhesion molecules.^[^ ^234^ ^]^ Finally, during the invasion step, the blastocyst invades through the stroma and glandular epithelium of the endometrium and implants.^[^ ^235^ ^]^ Implantation requires a fully competent blastocyst and a receptive‐state endometrium,^[^ ^236^ ^]^ which is a physiological and phasic state during the mid‐luteal phase of ≈48 h, termed the window of implantation (WOI). This window is characterized by molecular, cellular and morphological changes that prime the endometrium for implantation.^[^ ^237^, ^238^ ^]^ Implantation relies on numerous factors, and any perturbation can lead to implantation failure.^[^ ^239^ ^]^ These include but are not limited to inappropriately developed endometrium due to anatomical, immunological and endocrine complications or an incompetent blastocyst.^[^ ^26^ ^]^ Understanding implantation physiology is crucial for advancing ART techniques, improving fertility treatments, and addressing developmental biology questions. However, ethical and practical challenges limit research involving humans due to inaccessibility of the in utero environment.^[^ ^240^ ^]^ Thus, an increasing number of studies are being conducted to develop in vitro models of implantation and its molecular and mechanical cues.^[^ ^241^, ^242^, ^243^, ^244^ ^]^ The majority of studies have focused on combining human embryos with human primary epithelial cells.^[^ ^245^, ^246^, ^247^, ^248^, ^249^ ^]^

ECM engineering has become pivotal for the development of organoids by providing essential structural and biochemical cues for growth and organization.^[^ ^250^, ^251^ ^]^ ECMs are generally categorized as either synthetic or natural. Synthetic matrices allow precise control over composition and stiffness, whereas natural matrices, such as Matrigel and Cultrex, contain many proteins, such as collagen I and IV, fibronectin, and laminin,^[^ ^252^ ^]^ and growth factors that mimic the native cellular environment, supporting rapid upscaling of the groundbreaking endometrial organoid cultures.^[^ ^253^, ^254^ ^]^ Endometrial organoids are 3D, gland‐like structures derived from endometrial epithelial cells that closely mimic the architecture and function of the in vivo endometrial epithelium. These self‐organizing systems retain key physiological features, including hormone responsiveness, which enable them to recapitulate dynamic changes observed throughout the menstrual cycle.^[^ ^255^, ^256^, ^257^ ^]^ Specifically, organoids respond to sex steroid hormones such as estrogen and progesterone by altering gene expression, cellular morphology (distinct ciliated and secretory cell populations; luminal, glandular and stromal cells), secretory activity (MUC1 production), and closely mirroring the proliferative and secretory phases of the endometrium. This makes them valuable in vitro model for studying reproductive disorders^[^ ^258^, ^259^ ^]^ and implantation‐related processes.

**Figure 5:**
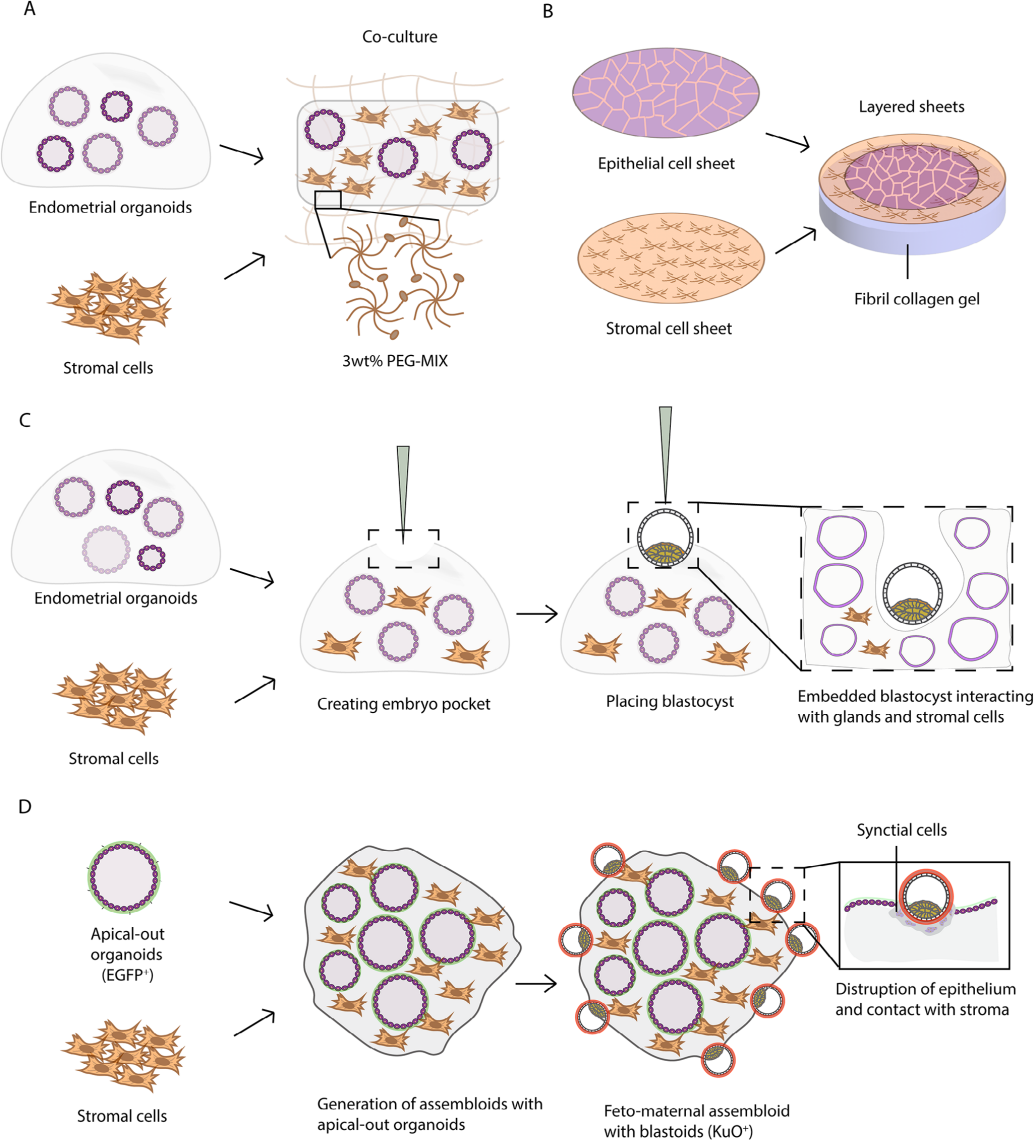
Advanced endometrial culture methods: from mimicking the endometrium to simulating implantation processes. A) Co‐culture of endometrial organoids with stromal cells in a tailored PEG‐MIX hydrogel for promoting the growth of both cell types. Adapted from.^[^ ^260^ ^]^ B) Creating epithelial and stromal cell sheets for co‐culture in fibril collagen gel to study the cell–cell interactions, as well as testing the effect of hormones. Adapted from.^[^ ^265^ ^]^ C) Co‐culture of endometrial organoids, stromal cells and blastocysts that were placed in “embryo pockets.” The insert highlights the interaction between the blastocyst and co‐culture in the gel. Adapted from.^[^ ^266^ ^]^ D) Generation of assembloids with apical‐out EGFP+ endometrial organoids and stromal cells. Once the assembloids were generated, KuO+ blastoids were co‐cultured and showed implantation‐like behaviors, including disrupting the epithelium and contacting with stromal cells. EGFP = Green fluorescent protein. KuO = Kusabira Orange. Adapted from.^[^ ^267^ ^]^

Combining our knowledge on native endometrial ECM and tissue engineering have deepened our understanding of endometrial biology. Recently, a group developed a poly‐(ethylene glycol) (PEG) hydrogel to support both endometrial organoids and stromal cells by creating a synthetic ECM using an 8‐arm PEG‐based hydrogel functionalized with GFOGER and PHSRN‐K‐RGD peptides, mimicking collagen and fibronectin adhesion moieties, respectively (**Figure** **5**A).^[^ ^260^ ^]^ This synthetic ECM was able to mimic different stages of menstrual cycle, with epithelial cells responding to progestin treatment and stromal cells exhibiting decidualization‐like behavior, including increased prolactin secretion.^[^ ^261^ ^]^ Decidualization, a crucial step of endometrial receptivity for implantation, is the molecular and functional transformation of the cells. To study inter‐reproductive organ crosstalk, Park et al. created a dual reproductive organ‐on‐a‐chip with two ovarian chambers and an endometrium chamber to study cross talk between these organs by combining engineered hybrid‐material matrices with microfluidic systems.^[^ ^262^ ^]^ The group used PDMS chips and embedded various polymers (agarose (0.6%, w/v), hyaluronic acid (2.5%, w/v), and type I collagen (2.5%, w/v)) to construct a porous endometrial layer, which matched human endometrial stiffness (18 kPa).^[^ ^263^ ^]^ In another study, natural ECM derived from decellularized porcine endometrium was mixed with the commercial synthetic PuraMatrix hydrogel in a 50:50 ratio to support the growth of endometrial organoids.^[^ ^264^ ^]^ The group thoroughly characterized the hybrid gel using rheology (for stiffness), SEM (for fiber content), and proteomics (for ECM‐related proteins) and showed that the gel promoted differentiation of the organoids by the significant upregulation of receptivity biomarkers. To increase anatomical complexity, in another study, rat endometrial tissue was cultured as 3D endometrial‐like sheets on a fibrin‐collagen gel base, creating a multilayered structure with epithelial and stromal cells, which are extracted from endometrial tissue (Figure 5B).^[^ ^265^ ^]^ Cell sheets preserve natural architecture and direct cell–cell interactions, which are crucial for mimicking the physiological conditions of the endometrium. This approach could lead to a more accurate representation of the cellular environment, supporting better tissue functionality and responses.

With advances in bioengineering, these sophisticated endometrial models now provide a dynamic platform to study not only cellular complexity but also critical processes like embryo implantation, enabling researchers to simulate and observe early‐stage embryo interactions within a controlled environment. In the Kagawa et al. study, the authors sought to model the initial steps of implantation using endometrial epithelial monolayers, derived from endometrial organoids, in conjunction with blastoids.^[^ ^202^ ^]^ The research group demonstrated that hormonally induced monolayers exhibited enhanced adherence of blastoids compared to non‐induced monolayers. Ak et al. used a microfluidic chip‐based platform to precisely quantify blastoid adhesion to endometrial monolayers by exposing them to a controlled step‐wise increasing flow rate.^[^ ^268^ ^]^ Luijkx et al. used this platform to show that monochorionic twins have increased adhesion potential compared to singleton blastoids.^[^ ^215^ ^]^ In another report, a co‐culture of gland‐like structures and stromal cells, named assembloids, were developed to test how decidual senescence may promote embryo implantation in the endometrium (Figure 5C).^[^ ^266^ ^]^ To test the response of the assembloids in terms of senescence, the group embedded human embryos by creating small pockets within the assembloids. The presence of the senescent decidual cells allowed the blastocyst to interact and expand. However, the continuous secretome production released by the senescent decidual cells led to the disintegration of the assembloids. This study highlights limitations that arise from a non‐dynamic platform, in addition to the absence of luminal epithelium for implantation studies. Advancing the assembloid model, Shibata et al. co‐cultured their assembloids model with blastoids (Figure 5D).^[^ ^267^ ^]^ This assembloids model consisted of apically out endometrial organoids (named AO–EMO), endometrial stromal cells and primary Human Umbilical Vein Endothelial Cells (HUVEC). Hormonally responsive assembloids were cultured with blastoids for 5 days, showcasing events at the feto‐maternal interface, including possibly cell fusion. Aforementioned studies focus on co‐culturing stromal cells with glandular epithelial cells including organoids but are lacking the luminal epithelium, which is crucial for modeling embryo implantation. To overcome that, Tian et al. generated human endometrium assembloids using an air‐liquid interface (ALI) method, which consists of both the glandular and luminal epithelium for the first time.^[^ ^269^ ^]^ This comprehensive study indicated that the assembloids faithfully replicated the in vivo endometrial anatomy, Young's modulus (450 Pa for top‐layer endometrium), cell composition, and gene expression profiles, including the WOI genes. Unfortunately, this study did not perform a functional assay to investigate embryo implantation.

Recent bioengineering advances have also extended to placental organoids, which together with endometrial organoids provide powerful platforms to model the maternal‐fetal interface and further implantation events.^[^ ^270^, ^271^, ^272^, ^273^ ^]^ In particular, trophoblast invasion, a critical step in early pregnancy involving deep interaction between invading placental cells and the remodeling of maternal ECM, relies heavily on finely tuned cell‐matrix signaling.^[^ ^274^ ^]^ Park et al. developed an “implantation‐on‐chip” with a fetal chamber, containing extravillous trophoblast (EVT) cells and a vascular chamber, containing endothelial cells, to mimic a further step of the implantation process, invasion through the maternal vessels.^[^ ^275^ ^]^ As the first step, the central channel was injected with a Collagen‐Matrigel mixture to mimic the 3D ECM of the endometrium, providing an environment for cell invasion from the side channels. The hydrogel scaffold's stiffness increased from 390.24 ± 146.25 to 826.26 ± 164.84 Pa, effectively mimicking the natural stiffness changes in human decidua and highlighting the crosstalk between endothelial cells and trophoblasts. To further investigate, the team tested the effect of decidualized stromal cells (DSCs) on EVT invasiveness by seeding DSCs in the hydrogel compartment and exposing them to hormonal induction. Importantly, the presence of DSCs reduced EVT invasiveness, as observed in controlled inhibition in vivo. The level of invasion is crucial to maintain, as too much invasion can lead to complications, and too little can result in conditions such as preeclampsia.

Collectively, the overarching goal of these models is to create a functional and personalized representation of the endometrium for application for medical application, serving as an additional diagnostic tool. Current diagnostic tools are restricted in their application spectrum, for instance the Endometrial Receptivity Array (ERA) test, which involves taking an endometrial biopsy during the presumed WOI. This procedure aims to assess the gene expression profile to identify the specific transcriptomic signature, ultimately providing a more precise timing for personalized embryo transfer (pET). However, as with many gene‐based methods, there are inconclusive results regarding which specific genes are associated with the WOI and the accuracy of their expression.^[^ ^276^, ^277^, ^278^ ^]^ These challenges are further complicated by the timing and method of sample collection and the mathematical models used.^[^ ^279^ ^]^ Therefore, there is an increasing need for broader and more functional assays, enabling ongoing, personalized study of endometrial physiology. Advanced microengineered platforms and in vitro models are well suited to fulfill this need, particularly in conjunction with functional endometrial assays that incorporate actual embryos or stem cell‐based embryo models. The integration of advanced biological models^[^ ^280^ ^]^ provides a deeper understanding of the intricate communication between maternal and fetal tissues, offering opportunities for developing personalized and precise ART methods. These models can reflect the complex structure of the endometrium by accurately mimicking its diverse cellular architecture, including the glandular and luminal epithelium, both critical for successful implantation and early pregnancy development. By replicating dynamic physiological conditions, such as hormonal fluctuations and changes in the uterine microenvironment, these systems enable the study of factors that influence endometrial receptivity and embryo development. Additionally, they serve as valuable platforms for testing therapeutic interventions aimed at improving implantation rates and pregnancy outcomes, allowing for the assessment of new drugs, hormones, or treatments in a controlled and reproducible environment.

**Figure 6:**
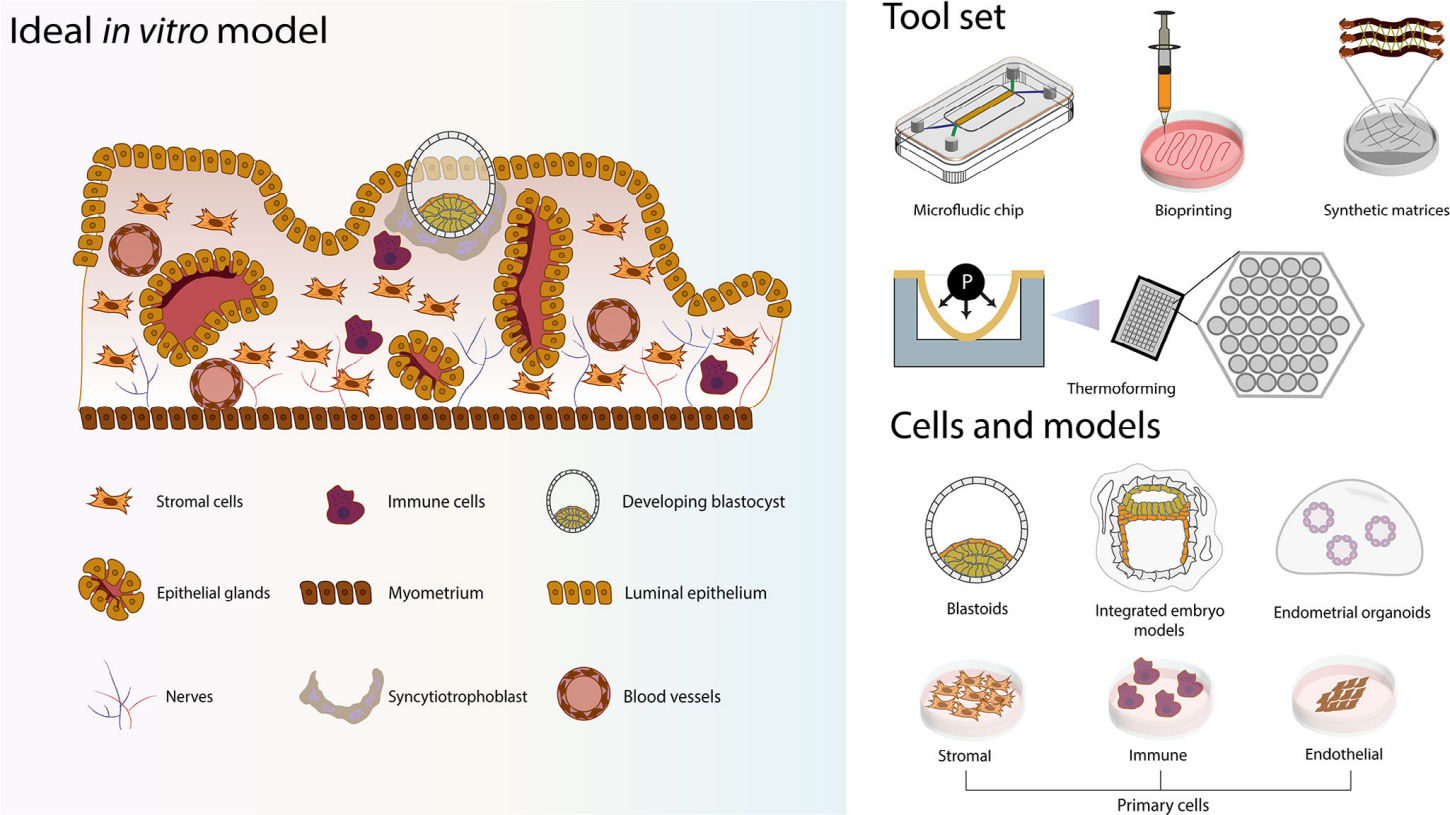
An ideal in vitro model of endometrium for embryo implantation with available tool sets, models, and cells.

An ideal in vitro model for reproductive medicine would incorporate advanced endometrial platforms integrated with blastoids or embryo‐like structures to replicate early human development and implantation (**Figure** **6**). This patient‐derived model would include multiple essential cell types: glandular and luminal epithelial cells to facilitate attachment, a stromal support layer to enable invasion, and endothelial and immune cells to support post‐implantation development. Integrating microfluidic systems would provide dynamic control over the culture environment, allowing precise delivery of nutrients, hormones, and growth factors while simulating perfusion‐based flow dynamics. These systems could mimic the cyclical changes of the uterine environment, further enhancing physiological relevance. Additionally, the incorporation of advanced imaging techniques and biosensors would enable real‐time monitoring of cellular behavior, embryo‐endometrium interactions, and molecular changes throughout the implantation process.

Clinicians could play a key role in refining and implementing such models by incorporating them into clinical trials to evaluate patient‐specific treatment responses. Their firsthand knowledge, gained through patient interactions, could help identify variability in implantation success and provide critical insights into atypical cases. By feeding this information back into the design of in vitro models, researchers could better capture patient‐to‐patient variability and improve model accuracy. This collaborative feedback loop between clinicians and scientists could enhance personalized ART approaches, ultimately translating laboratory research into practical clinical applications.

The left panel illustrates a representative in vitro model of the human endometrium, depicting key cellular and structural components, including the luminal epithelium, glandular epithelium, stromal and immune cells, myometrial layer, vasculature, an implanting blastocyst, and emerging syncytiotrophoblast. A diverse tool set is currently available to engineer these models, including microfluidic devices, bioprinting technologies, synthetic matrices, and thermoformed microwells. Cellular components and model systems include blastoids, integrated embryo models, endometrial organoids, and primary human cells such as stromal, immune, and endothelial populations, enabling the reconstruction of a physiologically relevant implantation microenvironment.

## 4 Discussion

This review aims to identify research gaps and possible improvements in the concurrence of reproductive medicine and bioengineering. While some aspects of ART could benefit from the integration of bioengineered platforms into routine practices, such as sperm sorting, the majority, including embryo culture, in vitro fertilization on these platforms, and oocyte handling, could not benefit yet. On the other side of the coin, the contribution of reproductive medicine to regenerative medicine and bioengineering cannot be underestimated. Knowledge and material, such as organs, tissues and cells, gained from patients are contributing tremendously to designing more tools and platforms. These technologies have the potential to streamline and upscale ART procedures, making them safer, more reliable, and potentially more cost‐effective. In addition, microfabrication strategies play a key role in forming and studying stem cell‐based embryo models that may improve our understanding of human development and feed regenerative medicine strategies. Future research should focus on overcoming current limitations, such as scalability, integration with bioanalytical systems, and ethical considerations, to fully realize the potential of these innovative tools in ART.

While microfluidics and similar platforms hold great promise for improving assisted reproductive technologies (ART), they come with significant limitations. Designing these platforms is often time‐consuming and expensive, requiring specialized expertise and equipment. Their complexity can also make them impractical for widespread use, limiting their accessibility to only a few advanced laboratories. Moreover, most studies utilizing these platforms are conducted with animal models, and there is a notable lack of research using human samples to validate their efficacy. This is why advanced in vitro models are essential. They serve as substitutes for human samples, providing a controlled environment to refine and test these platforms more effectively. By using in vitro models, researchers can bridge the gap between current technological capabilities and the biological complexities of human tissues. These models are essential for advancing our understanding of implantation biology and improving the success of ART, all while reducing the need for real human samples until the new technologies are fully validated. Finaly, it is imperative to acknowledge the current ethical limitations surrounding reproductive technologies and ensure that scientific advancements are governed ethically for the greater benefit of society. Smooth transitions between these aspects of research and application will facilitate the seamless integration of cutting‐edge techniques into clinical practice, ultimately improving outcomes for individuals seeking reproductive assistance.

## Conflict of Interest

The authors declare no conflict of interest.
